# Sustainable Production of *Ajuga* Bioactive Metabolites Using Cell Culture Technologies: A Review

**DOI:** 10.3390/nu15051246

**Published:** 2023-03-01

**Authors:** Elena Popova, Maria Titova, Marat Tynykulov, Rano P. Zakirova, Irina Kulichenko, Olga Prudnikova, Alexander Nosov

**Affiliations:** 1 K.A. Timiryazev Institute of Plant Physiology of Russian Academy of Sciences, Botanicheskaya 35, 127276 Moscow, Russia; 2 Faculty of Natural Sciences, L.N. Gumilyov Eurasian National University, Munaytpasov 13, Astana 010000, Kazakhstan; 3 S.Yu. Yunusov Institute of the Chemistry of Plant Substances, Academy of Sciences of the Republic of Uzbekistan, M. Ulugbek 77, Tashkent 100170, Uzbekistan; 4 Biology Faculty, M.V. Lomonosov Moscow State University, 119234 Moscow, Russia

**Keywords:** 20-hydroxyecdysone, anthocyanins, biomass accumulation, callus, elicitation, iridoids, phytoecdysteroids, suspension cell culture, turkesterone

## Abstract

The genus *Ajuga* (Lamiaceae) is rich in medicinally important species with biological activities ranging from anti-inflammatory, antitumor, neuroprotective, and antidiabetic to antibacterial, antiviral, cytotoxic, and insecticidal effects. Every species contains a unique and complex mixture of bioactive metabolites—phytoecdysteroids (PEs), iridoid glycosides, withanolides, neo-clerodane terpenoids, flavonoids, phenolics, and other chemicals with high therapeutic potential. Phytoecdysteroids, the main compounds of interest, are natural anabolic and adaptogenic agents that are widely used as components of dietary supplements. Wild plants remain the main source of *Ajuga* bioactive metabolites, particularly PEs, which leads to frequent overexploitation of their natural resources. Cell culture biotechnologies offer a sustainable approach to the production of vegetative biomass and individual phytochemicals specific for *Ajuga* genus. Cell cultures developed from eight *Ajuga* taxa were capable of producing PEs, a variety of phenolics and flavonoids, anthocyanins, volatile compounds, phenyletanoid glycosides, iridoids, and fatty acids, and demonstrated antioxidant, antimicrobial, and anti-inflammatory activities. The most abundant PEs in the cell cultures was 20-hydroxyecdysone, followed by turkesterone and cyasterone. The PE content in the cell cultures was comparable or higher than in wild or greenhouse plants, in vitro-grown shoots, and root cultures. Elicitation with methyl jasmonate (50–125 µM) or mevalonate and induced mutagenesis were the most effective strategies that stimulated cell culture biosynthetic capacity. This review summarizes the current progress in cell culture application for the production of pharmacologically important *Ajuga* metabolites, discusses various approaches to improve the compound yield, and highlights the potential directions for future interventions.

## 1. Introduction

The genus *Ajuga* of the family Lamiaceae comprises 80 accepted species and about 135 taxa (including subspecies and varieties) of annual and perennial herbaceous flowering plants, and is widely spread throughout the temperate and subtropical regions of Europe, Asia, Australia, North America, and Africa [1,2]. Many of *Ajuga* species have medicinal value and have been used for centuries in folk medicines to treat various disorders, including fever, hypertension, hyperglycemia, pneumonia, acute and chronic pharyngitis, joint pain, etc. [3,4,5]. Popular traditional remedies are made of *A. decumbens* Thunb., *A. bracteosa* Wall. ex Benth (accepted name *Ajuga integrifolia* Buch.-Ham.), *A. nipponensis* Makino, *A. ciliate* Bunge, *A. reptans* L., *A. turkestanica* (Regel) Briq., and several other species. Extracts and purified compounds from *Ajuga* spp. were reported to exhibit diverse biological effects, such as anti-inflammatory, antitumor, neuroprotective, antibacterial, antivirus, cytotoxic, and insecticidal activities [6,7,8].

Both roots and aerial parts of *Ajuga* plants are rich with biologically active molecules, including phytoecdysteroids (PEs), iridoid glycosides, withanolides, neo-clerodane terpenoids, a diversity of flavonoid and phenolic compounds, essential oils, and other metabolites with high therapeutic potential that are still being researched [2,5]. Special interest is focused on PEs, which have been identified as “natural anabolic and adaptogenic agents” in mammalians [9,10,11,12,13] and are widely used as active ingredients in bodybuilding and athlete dietary supplements [14]. PEs also have a potential role as a supporting treatment during therapy for chronic health conditions, such as arthritis, diabetes, and nervous system disorders, in wound healing, and as cosmetic ingredients [11,15]. Natural plant protection is another potentially interesting application of PEs-containing products due to their proven anti-feeding activities [16].

Currently, the majority of commercially important *Ajuga* metabolites are derived from plants, and therefore face the common problems associated with wild plant harvesting and field production: variable yield depending on the geographic origin, season, and environmental factors, low content of the desired compounds in the dried plant biomass, and limited availability of wild plant resources [17,18]. Plant and cell in vitro cultures may be used as an alternative means of producing vegetative biomass rich in pharmaceutically active compounds, including PEs [19,20,21]. Compared to shoot culture, production of cell suspensions in bioreactors has the advantages of automation and upscaling of the cultivation processes from laboratory to industrial volumes, and yield standardized batches of vegetative biomass with predetermined characteristics [22,23]. The common problem of low content of the desired compounds in cell biomass can be addressed through biotechnological strategies, such as optimization of culture conditions, elicitation, genetic engineering, or targeted selection of cell lines, with metabolism shifted towards increased production of the specific metabolites [24,25]. In this review, we summarize the outcomes of using cell culture technologies for the production of *Ajuga* bioactive metabolites, particularly PEs, including culture initiation and optimization of culture conditions; present strategies to improve the compound yield, and discuss the perspective of these approaches for practical applications.

## 2. Biologically Active Compounds of Genus *Ajuga* and the Need for Their Biotechnological Production

The biologically active ingredients of *Ajuga* plants belong to several main chemical groups: phytoecdysteroids, iridoids (iridoid glycosides), withanolides, neo-clerodane di- and triterpenoids, sterols, and a large range of flavonoid and phenolic compounds [2,26]. Chemical profiles of different *Ajuga* species as well as the biological activities of their extracts and isolated compounds have been thoroughly reviewed [2,4,5,6,7,8]. Bioactive components have been found in nearly all parts of the plants, including leaves, stem, roots, and flowers [2,8], although both their composition and the amount vary greatly among plant organs and in response to changing environmental conditions [17,18,19,27]. Table 1 presents, without pretention to completeness, the results of biochemical and biological studies of medicinally valuable *Ajuga* species, which were further explored for cell culture development. Among those, *A. bracteosa*, *A. reptance,* and *A. turkestanica* were the most intensively researched compared to *A. genevensis* L., A. *multiflora* Bunge, and *A. chia* (accepted name *Ajuga chamaepitys subsp. chia* (Schreb.) Arcang.), and very few or no studies are available for *A. lobata* D. Don (not included in Table 1).

### 2.1. Phytoecdysteroids and Their Role in Plants and Beyond

*Ajuga* spp. are particularly valued as natural producers of phytoecdysteroids, ecdysteroids (ECs) of plant origin [2,5,10]. Ecdysteroids are steroidal hormones that regulate insect development, molting, and metamorphosis [69,70,71]. The first ECs, ecdysone and 20-hydroxyecdysone (20E), were discovered in the 1950–1960s in arthropods [72,73]. Since then, ECs have been found in animals, plants, and fungi; some of them, such as 20E and ecdysone, are common among plant and animal kingdoms [15,69]. The Ecdybase [http://ecdybase.org (accessed on 23 November 2022)], the publicly open database of chemical structures, spectroscopic data, biological activities, and occurrence of ECs, currently contains 554 different compounds, compared to about 300 records available in 2009 [16]. New compounds including ECs are being constantly discovered in *Ajuga* spp. [64,65]. Interestingly, plants possess a larger diversity of ECs than arthropods where they were first discovered [21]. Arif et al. [15] stated that over 500 PEs have been identified in more than 100 terrestrial plants; however, PEs distribution in plant world is uneven. Several plant taxa are known to accumulate high levels of PEs, building up to 2%–3% on a dry weight (DW) basis [15,16]. These include plants of the genera *Ajuga, Achyranthes*, *Cyathula*, *Serratula*, *Silene*, *Podocarpus*, *Vitex*, *Pfaffia*, and some others. It is expected that recent advances in analytical methods and the targeted screening of the growing number of taxa may lead to the detection of PEs in a larger array of plant species [16].

PEs belong to the triterpenoid chemical group, and the majority of them have a polyhydroxylated four-ringed skeleton with a sidechain at C-17. The huge diversity of PEs is due to the different numbers of C-atoms (24C to 29C); the variation in the position, number, and orientation of hydroxyl and oxo groups; the number of conjugating groups, and the fact that the conjugating moiety can be polar or non-polar [70]. Structural variations via etherification, esterification, oxidation, amination, fluorination, and alkylation amplifies the diversity of PEs [15,16]. In addition, PEs in plants may exist in free-state or in conjugated forms, such as glycosides or esters [15].

The role of PEs in plants is not yet completely understood. It is proposed that they play a defensive role against insect and nematode attacks, as well as physiological functions yet to be discovered [2,10]. Under biotic and abiotic stresses, PEs act to promote the plant’s antioxidant system. Their crosstalk with phytohormones, including cytokinins, auxins, brassinolides, and jasmonate has been also proposed [15], although PEs per se do not seem to possess phytohormonal activities. Some PEs demonstrate allelochemical functions, i.e., they suppress the growth of other competitive plants or microbes when released into the environment [74].

PEs biosynthesis in plants, its regulation, and the mechanisms of PEs distribution between organs have been reviewed [15,16,21,70], although the current knowledge of these processes in still incomplete. As triterpenoid compounds, most PEs are synthesized mainly through the mevalonate (MVA) pathway in the cytosol, and have lathosterol and cholesterol as common precursors. Cholesterol is further converted to ecdysone and 20E through 7-dehydrocholesterol intermediate. However, the detailed mechanism as well as the location of PEs synthesis may differ between plant species. For example, lathosterol was considered the main precursor of PEs in *Spinacia oleracea* [75]. Hairy roots of *Ajuga reptans* were reported to use an alternative biosynthetic pathway for PEs synthesis, which does not involve 7-dehydrocholesterol as an obligatory intermediate [76]. The conversion of C28- or C29-sterols into the corresponding C28- and C29- ecdysteroids and the use of hydroxylated cholesterol derivatives as precursors of ecdysone and 20E have been also reported [16].

PEs are the main reason for the frequent use of *Ajug*a in traditional medicines and possess a large spectrum of biological and pharmacological activities, from antimicrobial (including antimalarial and antifungal) to anti-inflammatory, antioxidant, and antitumor (Table 1) effects. Anti-feeding and insecticidal effects reported for several PEs correlate well with their proposed role as the components of plant protective systems. In mammals, including humans, PEs demonstrate a wide range of pharmacological effects, mostly positive for the organism [11,14,77]. Data on neuroprotective and anabolic activities of some PEs suggest their great potential for pharmacology [9,11]. Over 47 different PEs have been reported in *Ajuga*, some of which were uniquely found only in this genus [78]. The majority of *Ajuga* species contain a mixture of PEs that commonly includes 1–3 major compounds (usually 20E, cyasterone and a species-specific PE) and a few minor PEs [16,26,78]. Turkesterone, found specifically in *A. turkesanica*, an endemic plant of Central Asia, has proven positive effects on muscle tissues, including improved signaling in aged skeletal muscles and an increase in protein synthesis in muscles and in liver, which underlies its commercial use as a popular food supplement [7,66,79].

### 2.2. Other Biologically Active Compounds of Ajuga Genus

Among other compounds found in *Ajuga* plants, iridoids, withanolides, and neo-clerodane terpenoids are promising pharmacological substances [80,81]. Iridoids, for example, exhibit a wide range of bioactivities, including cardiovascular, anti-inflammatory, antispasmodic, antitumor (including antiangiogenic), antiviral, neuroprotective, and immunomodulatory effects [82,83,84]. Reported pharmacological activities of withanolides include, among others, antitumor, immunomodulatory, antibacterial, anti-inflammatory, antiarthritic, and central nervous system effects [80,85]. Neo-clerodane diterpenoids ajugarin I and lupulin A, iridoids reptoside, and 6-deoxyharpagide, and withaferin A were thought to be responsible for the anti-arthritic activity of *A. bracteosa* extracts in an albino rat model [28]. Aerial part extracts of *A. genevensis*, *A. chamaepitys,* and *A. laxmannii* containing iridoids, polyphenols, and flavonoids exhibited antitumor and anti-inflammatory activities in murine colon carcinoma and melanoma cell lines [43]. Two iridoids, asperulosidic acid and deacetyl-asperulosidic acid, identified in the extract of *A. chamaepitys*, were thought to be responsible for the extract activity against ulcerative colitis [50]. Extracts from *A. genevensis* and *A. reptans* that contained iridoids, sterols, polyphenolic, and flavonoid compounds demonstrated antimicrobial, anti-inflammatory, and antioxidant activities [44]. Flavonoid and phenolic compounds as well as amino acids were detected in a crude extract of *A. bracteosa* with high activity against the hepatitis C virus [29]. Anthocyanins found in many *Ajuga* species lower the risk of cardiovascular disease, diabetes, arthritis, and cancer, possibly due to their anti-oxidant and anti-inflammatory activities [86,87]. Phenolic glycosides and the α- and β-pinene-rich essential oils were found in *A. chamaepitys* extracts with high free-radical scavenging properties [51]. Other compounds found in *Ajuga* species, such as luteolin, ferulic acid, chlorogenic acid, caffeic acid, and others (Table 1) are known to exert a wide array of biological activities including antioxidant, anti-inflammatory, antimicrobial, and anticancer effects.

### 2.3. Cell Culture as an Alternative Option for Sustainable Production of Phytochemicals

Wild *Ajuga* plants remain the main source of specific metabolites of this genus, which often leads to over-exploration of their natural recourses. Moreover, PEs content in wild plants differs significantly between organs, and shows high seasonal and geographical variations [18,19,27,52]. These factors limit availability and reduce the effectiveness of PE production from natural plant sources. Chemical synthesis of PEs derivatives has been reported [88], but is currently considered unprofitable, due to the molecule complexity and high structural diversity of PEs found in plants that are difficult to mimic in the laboratory [16]. Moreover, PEs may act in synergy with other secondary metabolites while performing their biological functions.

To overcome those issues, cell cultures may be developed as a renewable and high-quality source of bioactive compounds and become a sustainable alternative to wild plant collection [21,89,90]. Production of bioactive compounds by cell cultures is of both scientific and practical interest. From the physiological viewpoint, plant cell culture is a population of constantly proliferating undifferentiated cells, and as such it represents a suitable model for investigating biosynthetic pathways and their regulation [22]. From the biotechnological perspective, plant cell culture grown under sterile and thoroughly controlled conditions on the surface (callus) or in liquid (cell suspension) nutrient medium offers multiple options to control the composition and quantity of the final product [23]. The most common strategies include manipulation with medium composition and culture conditions, chemical elicitation, and precursor feeding, as well as gene engineering and mutagenesis to develop highly productive cell lines [24,89]. Dried biomass or crude extracts can be utilized as such, or for isolation and purification of individual compounds.

**Figure 1:**
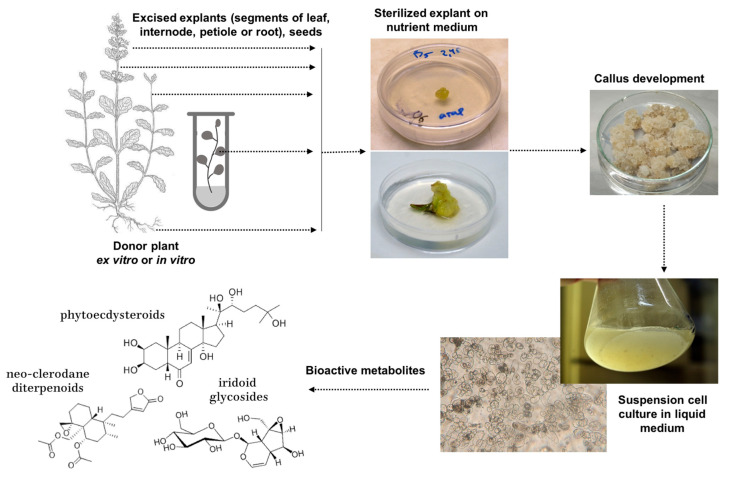
Schematic process of callus and suspension cell culture induction from the plant for the production of bioactive *Ajuga* components; phytoecdysteroids (20-hydroxyecdysone), neo-clerodane diterpenoids (ajugarin I), and iridoid glycosides (aucubin) are given as representative of their chemical classes. Photographs © IPPRAS. *Ajuga* plant drawing: Electronic Atlas of the Flora of British Columbia [eflora.bc.ca].

The major bioactive compounds of *Ajuga* belong to the terpene, polyphenolic, and flavonoid classes of secondary metabolites that can be synthetized and accumulated in significant amounts in the undifferentiated cell culture [90,91,92]. The process is schematically illustrated in Figure 1. The major challenges are usually related to the slow growth of the cell culture, the low content of target compounds, and the instability of the metabolite production over time.

## 3. Cell Culture Establishment in Different *Ajuga* Species

At the date of this review, undifferentiated cell cultures have been reported for eight taxa of *Ajuga* genus: *A. bracteosa, A. chia*, *A. genevensis*, *A. lobata*, *A. multiflora*, *A. pyramidalis*, *A. reptans,* and *A. turkestanica*. Among them, *A. pyramidalis* and *A. chia* are currently considered synonyms of *A. genevensis* and *A. chamaepitys* [1], respectively, but in this review, we retained the taxon name from the original publications to allow easy data-to-reference tracking.

Cell cultures could be successfully established using virtually all parts of the plants, including seeds, roots, leaves, petioles, internodes, and ovary tissues (Figure 1, Table 2). In some cases, leaves or hypocotyles of in vitro plantlets were used as explant sources for higher sterility [93,94,95]. Nutrient media with Murashige and Skoog mineral salt base was the most commonly used, followed by Gamborg (B5) medium applied for developing cell cultures of *A. reptans* and *A. turkestanica,* and a Woody Plant Medium that was used for *A. pyramidalis* (Table 2). The medium for cell culture induction and maintenance usually contained both auxins and cytokinins in concentrations below 2 mg/L. The most used were combinations of cytokinin 6-benzylaminopurine (BA) with auxins α-naphthalene acetic acid (NAA) and 2,4-dichlorophenoxyacetic acid (2,4-D), while thidiazuron (TDZ) was also effective for cell culture induction in *A. bracteosa* and *A. turkestanica* (Table 2).

In *A. bracteosa*, maximum callus production was observed from leaf explants inoculated on MS medium with 5.0 mg/L BA followed by petiole explants on medium with 2.0 mg/L BA + 3.0 mg/L indole-3-acetic acid (IAA) and internodal explants on medium with 2.0 mg/L BA + 5.0 mg/L NAA [96]. In another study using the same species, 28 variants of five growth regulators (BA, 2,4-D, NAA, IAA, and indole-3-butyric acid [IBA]) applied individually or in combination were tested, and the highest callus formation rate (92.32%) was observed on MS medium with 2 mg/L BA and 1 mg/L 2,4-D [95].

Ali et al. [94] investigated the effects of growth regulators and light regimes on callus induction from sterile hypocotyls of *A. bracteosa*. Among growth regulators (kinetin, BA, 2,4-D, and IBA) and exogenous elicitors methyl jasmonate (MeJ) and phenylacetic acid (PAA), 1.0 mg/L BA induced maximum accumulation of callus (11.3 g/L) and suspension (13.2 g/L) cell biomass [94]. The most actively growing callus was obtained in darkness, whereas the slowest callus growth was recorded under continuous light. In the same species, Din et al. [97] explored the effects of the photoperiod during 4 weeks of callus induction, and found that darkness for two weeks followed by two weeks of light provided the highest callus initiation (90%) and biomass formation (5.6 g/L fresh weight [FW]). Among monochromic lights, callus induction frequency was highest (90%) under yellow light, while the most intensive biomass accumulation (28 g/L) was observed under red light, followed by yellow light (24 g/L) and control (white) light [97].

Suspension cell cultures were normally established by suspending friable callus in liquid medium, followed by selection of proliferating cell fractions during the next passages to a fresh medium, and in some cases, modification of growth regulators (Table 2). The established cell suspensions showed classical S-shape growth curves with subculture cycles lasting from 15 to 25 days depending on species and cultivation conditions [93,94,99,100,104]. Maximum culture productivity in terms of biomass concentration per L medium was recorded at the end of the exponential growth phase (day 9–12, specific for every cell culture) and varied from 0.65 g/L for *A. lobata* and *A. multiflora* [99,100] to 11.5–13 g/L for *A. reptans* [111,112], 13 g/L for *A. bracteosa* [94], and up to 17 g/L for *A. turkestanica* [113]. High cell viability and mitotic activity were also reported for cell suspension cultures of *A. turkestanica* and *A. reptans* [101,113].

Once established, callus and suspension cell cultures demonstrated a relatively stable growth and could be researched extensively during the following years. For example, the growth index of *A. turkestanica* callus on the second year of cultivation was as high as 9.3 [103]. Callus culture of *A. reptans* even showed an increase in growth index upon prolonged cultivation for 6 years (from 10.2 to 12.43 based on DW), possibly as a result of a constant auto-selection of cells with high proliferative ability [101]. Specific growth rates of callus (0.13) and suspension (0.35) cell cultures of this species also remained stable during 6 years of continuous cultivation [101].

## 4. Phytoecdysteroids in *Ajuga* Cell Cultures and Strategies for Their Enhanced Production

The occurrence of PEs in plant cell cultures was first demonstrated in the 1970s in the callus of *Achyranthes fauriei* [114] and *Trianthema portulacastrum* [115]. Later on, callus and suspension cell cultures of *Serratula coronata*, *Chenopodium album*, *Vitex glabrata,* and some other species were developed as potential biotechnological sources of PEs [16,101,116,117,118].

In *Ajuga* genus, biosynthesis of PEs was reported for cell cultures of *A. genevensis*, *A. lobata*, *A. multiflora,* and *A. turkestanica* (Table 3). In the majority of studies, the main detected PE compound was 20-hydroxyedysone, with concentrations varying from trace amounts to 12.75 mg/g (Table 3). Another well-known PE, turkesterone, was identified in the cell cultures of *A. turkestanica* [104,105,119,120]. Cell cultures of *A. reptance,* in addition to 20E, was also capable of producing polypodine B, 29-norcyasterone, ajugalacton, and ajugasterone [18,121]. In order to improve a relatively low content of PEs in several studies, various strategies were implemented, including chemical elicitation, altering the combination of growth regulators, changing light quality and photoperiod, and mutagenesis. These strategies are discussed below.

### 4.1. Nutrient Medium Composition

The relationship between suspension cell culture growth, its biosynthetic ability, and the uptake of nutrients from the medium was investigated in *A. multiflora* and *A. lobata* [99,100]. Accumulation of 20E in both cultures negatively correlated with the electric conductivity of the medium. As with cell cultures of many other species, phosphate was a limiting factor for cell biomass accumulation, and its concentration was depleted to almost zero after 11–13 days of culture when nitrate, ammonium, and sucrose were still available. In both cell cultures, the 20E concentration was maximized on days 5–6, which corresponded to the middle of the exponential growth phase [99,100].

In *A. reptans* callus culture, the supply of extra MnSO_4_ to the nutrient medium up to the final concentration of 2.5 mM increased both growth index (up to 5.36) and PEs content [121]. The amount of 20E was increased by 1.9 mg/g compared to control, 29-norcyasterone by 0.6 mg/g, and ajugalactone by 0.1 mg/g, while polypodine B was found in trace amounts. A further increase in Mn concentration to 12.5 mM stimulated the production of polypodine B by 0.7 mg/g but decreased the content of other PEs. A stimulative effect of MnSO_4_ on PEs production was also observed in plants of the same species after watering or spraying on leaves [121].

### 4.2. Growth Regulators in Culture Medium

Supplementation with abscisic acid (ABA) in the range of 0.1–0.5 mg/L in the suspension cell culture of *A. lobata* resulted in the increase in 20E content associated with high activities of the anti-oxidative system enzymes superoxide dismutase (SOD), catalase (CAT), and peroxidase (POD), as well as phenylalanine ammonia lyase (PAL), the key enzyme in the biosynthesis of polyphenol compounds [123]. However, the treatment killed the cells within a few days [123]. Later, the authors reported enhanced accumulation of 20E (up to 7 mg/g) 48 and 72 h after treatment with 0.15 mg/L ABA [125], but as in the previous study, the cell became brown after 96 h of ABA treatment. The transcriptome analysis of the cells after addition of 0.15 mg/L ABA revealed a total of 154 genes with changed expression (99 up-regulated and 55 down-regulated). Among the secondary metabolite pathways, differently expressed genes were reported for terpenoid backbone biosynthesis (6 genes), steroid biosynthesis (5 genes), and steroid hormone biosynthesis (6 genes). Pyrimidine and nitrogen metabolism genes were also among the most affected by ABA treatment [125].

Zakirova et al. [120] reported the production of about 100 callus and suspension cell lines of *A. turkestanica* from leaf explants of wild plants, followed by screening of the selected cell lines for the presence of 20E and turkesterone. The most productive cell lines accumulated 20E in concentrations of 2.0–2.5 mg/g. Higher 20E and turkesterone yield were observed in cell culture grown on MS medium with NAA (0.1 mg/L) and BA (0.05 mg/L) or kinetin (0.1 mg/L), while the presence of 2,4-D suppressed cell growth and resulted in brown and watery non-productive callus.

### 4.3. Elicitation and Precursor Feeding

In order to increase 20E biosynthesis in the cell cultures of *A. multiflora* and *A. lobata*, its precursors L-phenylalanine (L-Phe), mevalonic acid (MVA), α-pinene, and terpineol were added to culture medium [99,100]. Monoterpene α-pinene and its derivative, a monocyclic terpene alcohol terpineol, may act as inhibitors of terpene synthesis, altering the synthesis pathway to increase the yield of steroid ketones [99]. Mevalonate is the main substrate of the MVA biosynthetic pathway, and L-phenylalanine, the essential amino acid, is one of the basic precursors in secondary metabolic pathways. Among these compounds, 5–10 mg/L MVA and 1 mmol/L terpineol were the most effective and enhanced the PEs production in *A. multiflora* cell culture from 1.82 mg/g (control without elicitation) up to 4–6 mg/g [100]. An amount of 6–12 mmol/L, α-pinene showed a moderate effect resulting in a ca. 1.5-fold increase in 20E content in *A. multiflora* [100]. NO stress induced by sodium nitroprusside (SNP) slightly promoted accumulation of 20E up to 2.87 mg/g [100].

In *A. lobata*, both biomass and 20E content were increased significantly (up to 12.75 mg/g) by the combination of the optimum concentrations of elicitors in one treatment [99]. This included α-pinene (50 μL/L) and MVA (10 mg/L) supplied at day 1 followed by SNP (80 μmol/L) added at day 7 of the cultivation cycle. When the same elicitors were applied solely, the highest 20E content (11.75 mg/g) was achieved in the presence of 10 mg/L MVA [99]. L-phenylalanine showed no significant effect on 20E content [99,100].

Elicitation with 10–50 µM/L MeJ improved accumulation of both cell fresh weight and 20E in *A. lobata* cell suspension; the highest 20E concentration reached 1.4 mg/g which was nearly fivefold higher than in control culture without elicitation [124]. Higher concentrations of MeJ (100–200 µM/L) suppressed cell growth but further improved 20E production to 3.5 mg/g [99].

Cheng et al. [104,127] studied the effects of MeJ and precursor feeding in leaf-originated cell cultures of *A. turkestanica* with the ability to produce a mixture of 20E, turkesterone, cyasterone, and cyasterone 22-acetate. The authors observed a significant increase in the biosynthesis of 20E and turkesterone following elicitation with 15 mg/L and 150 mg/L MVA [127]. In the later work, the authors reported that the average concentration of 20E in 10- to 15-month-old suspension cultures was 6.91 µg/g, while turkesterone, cyasterone, and cyasterone 22-acetate were only found in trace amounts [104]. The content of PEs was not affected by supplementing precursors (MVA, cholesterol, and sodium acetate). Elicitation with 125 or 250 µM MeJ resulted in non-significant increases in 20E content, up to 23.6 and 14.7 l µg/mg extract, respectively [104].

Presumably, among all treatments reviewed, MeJ (50 to 125 μmol/L) and MVA (10 mg/L) were the most effective in improving PEs biosynthesis in *Ajuga* cell cultures and could be recommended for further applications.

### 4.4. Induced Mutagenesis

Zakirova et al. established callus and suspension cell cultures of *A. turkestanica* capable of producing both ecdysterone and turkesterone [105,113]. The culture derived from ovaries of the wild plant produced in the first passage 0.1% ecdysterone and 0.032% turkesterone, but the content of both compounds decreased over time [128], and the 20E content dropped down to 0.029–0.035% [105,113]. In order to increase the yield, the original cell culture was treated with the mutagen N-nitroso-N-methylurea (N-NMU) [119]. The dose of 8 mM for 1 h was sublethal but produced a cell strain with increased 20E yield (0.2% vs. 0.01% in the original cell culture). The culture also retained the ability to produce turkesterone at ca. 0.004% [128] and iridoids harpagide and 8-O-Ac-harpagide [119].

### 4.5. Phytoecdysteroid Production and Culture Growth Phases

The production of PEs in suspension cell cultures was closely related to growth phases in the subcultivation cycle. The 20E content in *A. lobata* suspension cell culture peaked during the second half of the exponential growth phase (day 6 of the cultivation cycle) and then remained stable until the end of the subcultivation (day 19) [99]. Similarly, in the suspension cell cultures of *A. multiflora*, the 20E production maximized at day 5 (the middle of the exponential growth phase) at 5.068 mg/gDW [100]. The biosynthesis of 20E in the suspension cell culture of *A. turkestanica* was first detected on day 7 (exponential growth phase), increased in the stationary phase (day 20), and slightly reduced again at the degradation phase (day 25) [113]. In a later report using a different cell culture of the same species, production of both 20E and turkesterone was higher on day 27–34 compared to day 21 of the culture cycle [120]. In *A. reptance*, the 20E content average over the subculture cycle of cell suspension ranged from 0.55% to 0.68% [101]. In general, maximum production of 20E in suspension cell cultures was associated with late exponential and stationary growth phases.

### 4.6. Stability of Phytoecdysteroid Content in Cell Cultures during Continuous Cultivation

Accumulation of 20E in the suspension culture of *A. pyramidalis* varied among subcultivations (passages): it increased during the first subcultivations reaching a maximum of ca. 4.3 mg/g at passage 5 then gradually declined until ca. 1.4 mg/g (almost threefold) by passage 15 [100]. Likewise, the 20E content in *A. lobata* suspension cell culture increased during the first three subcultures and maximized at 5.11 mg/g at passage 4, followed by gradual decline to 1.5 mg/g at passage 15 [99].

In both the original and mutated cell lines of *A. turkestanica*, the contents of 20E and turkesterone decreased over the course of 4 years [128]. A twofold decline in 20E content was observed in both strains during the first year of cultivation (from 0.2 to 0.1% in mutated culture). After 4 years, 20E was only found in trace amounts (0.003%). Turkesterone was even more unstable; only traces of this compound were detected after 1–2 years of continuous cultivation.

By contrast, accumulation of 20E in callus culture of *A. reptans* remained stable after 6 years of continuous cultivation [101].

## 5. Anthocyanin Production in *Ajuga* Cell Cultures

The accumulation of anthocyanins, commercial food colorants and pharmacologically important antioxidants by plant cell cultures has been researched as a promising tool for their large-scale sustainable production. At different times, cell cultures of various species were tested for this purpose, including *Daucus carota*, *Aralia cordata*, *Vitis vinifera*, *Hibiscus sabdariffa*, *Euphorbia millii* and even *Populus* (*P. maximowiczii x P. nigra*) [129,130,131,132,133,134]. Another class of pigments, betacyanins, were obtained from the cell culture of *Beta vulgaris* [135]. The highest anthocyanin yield of 13% dry weight was achieved by Yamakawa et al. [136] in *Vitis* cell suspension culture. Despite the fact that most of these pilot productions were stopped because of high production cost or never went beyond the laboratory scale, these earlier studies revealed that not only the content, but also the composition and stability of the produced pigments were critically important.

### 5.1. Anthocyanins in Ajuga Cell Cultures–General Considerations

Among *Ajuga* species, *A. pyramidalis* Metallica Crispa was considered a potential candidate for anthocyanin production in cell culture due to its purple foliage. Madhavi et al. [137] induced callus and suspension cell cultures of this plant using Woody Plant Medium supplemented with PVP, 2.26 μM 2,4-D, and 3.49 μM kinetin. Production of anthocyanins was induced by increasing the carbohydrate level in the medium to 50 g/L and placing the culture under a photosynthetic photon flux of 55 μmol/m^2^s. Under optimized conditions, suspension cultures produced 41–42 mg of anthocyanins/100 gFW as compared to 5–7 mg in callus cultures, and 10–12 mg/100 gFW in the leaves of greenhouse plants [137]. Cyanidin was the major aglycon in the acid hydrolysate of the total anthocyanins [137]. The structure of the major pigment in the suspension cultures of *A. pyramidalis* was determined to be 3-*O*-(6-*O*(*E*)-ferulyl)-2-*O*-[(6-*O*(*E*)-ferulyl)-ß-D-glucopyranosyl-ß-D-glucopyranosyl]-5-*O*-(6-*O*-malonyl)-ß-D-glucopyranosylcyanidin [137].

The developed cell culture was also able to produce ferulic acid, and its accumulation positively correlated with pigmentation. The content of both soluble ferulic acid and anthocyanins reached its maximum in the middle of the exponential growth phase of the cell culture cycle (around day 8) and remained stable until day 15 through the stationary growth phase and degradation [93]. The maximum content of ferulic acid in the cell culture reached 138 mg/100 gFW, which was higher than in the leaves of the greenhouse-grown plants (24 mg/100 gFW) [93].

The cell cultures originating from the flower and leaf of *A. reptans* produced acylated anthocyanins [111,112,138]. The major anthocyanins of the flower and flower-induced cell cultures were determined as delphinidin 3-(di-*p*-coumaroyl)sophoroside-5-malonylglucosides and cyanidin 3-(di-*p*-coumaroyl)sophoroside-5-malonylglucosides, respectively [139]. Later, Terahara et al. [140] isolated individual anthocyanins from *A. reptans* flowers and flower-derived cell cultures, and their structures were identified as delphinidin 3-(*p*-coumaroyl-feruloyl)sophoroside-5-malonylglucoside, delphinidin 3-(diferuloyl)sophoroside-5-malonylglucoside, and cyanidin 3-(di-*p*-coumaroyl)sophoroside-5-glucoside. The other two compounds detected in the cell culture were tentatively identified as delphinidin 3-(diferuloyl)sophoroside-5-glucoside and cyanidin 3-(feruloyl-*p*-coumaroyl)sophoroside-5-malonylglucosi. The authors also identified two anthocyanin acyltransferases that produced acylated cyanidin- and delphinidin-based anthocyanins [141].

Light increased the anthocyanin accumulation in callus cells (2.5–3%DW), but the pigments were also produced in the dark (1%) [112]. Anthocyanin production was associated with the activity of PAL, the first enzyme of the anthocyanin biosynthesis route, which was generally higher in anthocyanin-producing callus lines [112].

Interestingly, in the initial reports on the *A. reptans* cell culture, suspension cells were less capable of producing anthocyanins (2% DW) compared to callus cultures (2–4% DW), and the production dropped further to 0.2% DW after 8–12 subculture cycles [111,112]. Later, however, a suspension cell line with stable production of anthocyanin over 5 years was reported and even tested for cultivation in a 2-l bioreactor [142]. These suspension cultures were heterogeneous and contained both pigmented and colorless cells. The authors also found that both content and composition of anthocyanins in various callus and suspension cell lines was changed after 5 years of continuous cultivation. In general, accumulation of delphinidin-based anthocyanins decreased in time. This tendency was more profound in the suspension cell culture, where they constituted less than 5% of total anthocyanins, while callus lines still produced more than 20% of these pigments [142]. The decrease in acylated anthocyanins was also notable. The author concluded that the accumulation of 50-substituted and acylated anthocyanins decreased during the transfer from solid culture to liquid culture.

### 5.2. Effect of Carbohydrate Source on Anthocyanin Production

The effects of various carbohydrate sources and growth regulators on growth and anthocyanin production in the cell lines was studied in *A. pyramidalis*. Anthocyanin accumulation was similar in cells grown on medium with sucrose, fructose, or glucose. Galactose enhanced anthocyanin accumulation while arabinose and lactose did not support cell growth [137]. This was in line with the report in *Daucus carota* cell culture where galactose enhanced both biomass and anthocyanin production [143]. On the contrary, growth and anthocyanin production in *A. reptans* grown on an equimolar mixture of glucose and fructose (1.5% each) were severely reduced compared to sucrose [112]. The authors also developed the cell culture that produced anthocyanins on medium with milk whey, using lactose as the only carbon source [138]. Increasing sucrose concentration in the medium from 30 to 50 g/L favored anthocyanin production [137]. In *A. reptans* calli, 2% of sucrose was optimum for the accumulation of anthocyanins [112].

### 5.3. Effect of Growth Regulators on Anthocyanin Production

Among different growth regulators tested, 2,4-D combined with kinetin resulted in higher growth rates but low anthocyanin level in *A. pyramidalis* [93]. The highest anthocyanin content was induced by combinations of IAA or NAA with zeatin. Gibberellic acid (GA_3_) in varying concentrations (up to 1000 μM) inhibited biomass accumulation and production of both anthocyanins and ferulic acid [93]. Similarly, growth regulators 2,4-D and GA_3_ reduced anthocyanin production in *A. reptans* and *D. carota* [112,144]. It is thought that negative effect of 2,4-D and GA_3_ was due to inhibition of chalcone synthase, the key enzyme in the anthocyanin biosynthetic pathway responsible for the synthesis of the C_6_-C_3_-C_6_ carbon skeleton of flavonoids [93].

### 5.4. Stability of Cell-Culture-Produced Anthocyanins

The stability of the anthocyanins produced in the cell culture of *A. pyramidalis* was assessed by exposing anthocyanin-containing extracts to constant radiation provided by fluorescent lamps (140 μmol/m^2^s), with regular measurements of the retained absorbance at 520 nm (see [137] for details). In this experiment, anthocyanins from the cell culture extract were considerably more stable under light exposure compared to those from the in vivo plant extract: only 28% of the cell culture-derived anthocyanins degraded on day 15 compared to 66% of anthocyanins derived from the in vivo plant extract. Even after 30 days of light exposure, the cell-derived extract retained over the half of the original absorbance, indicating the presence of the pigments.

The results of these reports suggest that cell lines, both callus and suspension, with high and stable accumulation production of anthocyanins can be selected under favorable conditions (sucrose or galactose as a carbon source and the combination of IAA or NAA with cytokinins as growth regulators). These cell cultures were capable of accumulating relatively light-stable pigments and retained their production for at least several years, although the composition of the anthocyanins could be shifted in the course of the long-term cultivation.

## 6. Other Biologically Active Compounds Produced in *Ajuga* Cell Cultures

Compared to PEs, little or no information is available regarding the possibility of producing withanolides, iridoids, and neo-clerodane diterpenoids in *Ajuga* cell cultures. A single publication reported that, in addition to 20E, iridoids harpagide and 8-O-Ac-harpagide were detected in the cell cultures of *A. turkestanica* together with phytoecdysteroids 20E and turkesterone after chemical mutagenesis induced with N-NMU, but they were absent in the original culture [119].

Several studies explored the accumulation of phenolics and flavonoids, volatile compounds, phenylpropanoid glycosides, fatty acids (FA), and polysaccharides in cell cultures of different *Ajuga* species (Table 4). In *A. bracteosa* cell culture, higher levels of total phenolic and flavonoid content as well as free-radical scavenging activity were recorded in callus cultures grown under continuous light, while the best callus biomass accumulation was achieved in darkness [94]. MeJ (0.5 mg/L) and PAA (1.0 mg/L) were most effective in increasing total phenolic and flavonoid content and free-radical scavenging activity in the suspension cell culture [94]. Elicitation with 0.5 mg/L MeJ also resulted in higher activities of SOD and POD enzymes in the cell culture compared to PAA and various growth regulators tested.

Similarly, 0.5 mg/L MeJ was most effective for increasing production of volatiles in *A. bracteosa* cell culture under both light and dark conditions [94]. High levels of the monoterpene hydrocarbons such as β-ocimene and myrtenal could be also found in the dark-grown cell cultures in the presence of 1.0 mg/L of PAA and BA.

A total of 29 volatile compounds were identified in the essential oil profiles of the *A. bracteosa* cell cultures [94]. The identified compounds consisted of monoterpene hydrocarbons such as β-pinene, β-ocimene, 1-terpinene-4-ol, caryophyllene, β-farnesene, oxygenated monoterpenes such as myrtenal, citronellyl acetate, and sesquiterpenes, such as caryophyllene oxide and β-elemene. The composition of volatile secondary metabolites was different between light and dark cultures. Sesquiterpene volatiles were mostly found in 16 h light/8 h dark-growth cultures, while cultures in darkness produced more monoterpene hydrocarbons [94].

Suspension cell culture of *A. reptans* with high content (4 g/L) of teupolioside, a biologically active phenylpropanoid glycoside, was reported by Di Paola et al. [146]. Several types of the teupoloside-rich dried extracts derived from the cell culture are commercially available as certified food supplement ingredients [https://abres.it/en/teupolioside/ (accessed on 20 November 2022)].

In *A. genevensis,* the content of neutral lipids in callus cultures of root origin was almost 1.5-fold higher than in the callus of leaf origin (0.9% versus 0.65%) and comparable to the leaves of the wild plants (1.05%) [145]. In *A. chia*, the content of neutral lipids in callus was 0.74–1.48% versus 1.29% in plant leaves. The major components of neutral lipids of all samples were sterols and their esters, free fatty acids and their esters, and triacylglycerides. Palmitic acid predominated among the saturated FA, while linoleic acid was the main unsaturated FA. Stearic, oleic, linolenic, and arachic FA were detected in callus cultures of both species [145]. In general, callus cultures contained more unsaturated FA than saturated, and the highest content of unsaturated FA was found in the callus of *A. chia*.

Polysaccharide content and composition were analyzed in callus cultures of *A. turkestanica* [126]. Water-soluble polysaccharides were quantitatively a major fraction, but pectin substances were also present. The main structural molecule was galactose followed by arabinose, while xylose and rhamnose were missing or present in small amounts [126]. Ethanol extracts of callus samples contained fructose, saccharose, and fructooligosaccharides.

## 7. Bioreactor Cultivation of *Ajuga* Cell Suspensions

There are few reports on bioreactor cultivation of *Ajuga* cell cultures. A suspension cell culture of *A. reptans* was grown for anthocyanin production in a 2-l bioreactor equipped with a marine impeller and aerated by the compressed air [142]. The ratio of 10 anthocyanins remained relatively stable in the course of the cultivation cycle (one bioreactor run). The anthocyanin content peaked near day 15, which corresponded to the culture transition from exponential to stationary growth phase. The final yield of anthocyanins was 0.68–1.7%, depending on the cell line, and the highest productivity varied from 9 to 18 mg anthocyanins per liter per day [112]. This was in general lower than the anthocyanin content in flasks (around 2.5%), potentially due to the additional stress during stirring and cell loss on the bioreactor walls and the stirrer surface [112]. Bioreactor cultivation of *A. reptans* cell culture for the production of teupolioside was mentioned by Di Paola et al. [146], but no further details were provided.

## 8. Biological Activities of Cell Culture Extracts and Individual Compounds

### 8.1. Antioxidant Activity

Aqueous extracts of *A. genevensis* callus cultures showed prominent antioxidant activity that was similar or higher than that of the intact plant. In this study, 10 μL of extract prepared from callus tissues of leaf and root origin showed antioxidant activity equal to 67.5 nM and 95.5 nM ascorbic acid, respectively, while the same amount of the intact plant extract had antioxidant activity equal to 65.5 nM ascorbic acid [98].

Both photoperiod and light spectrum had a significant impact on the antioxidant activity of callus extracts of *A. bracteosa* measured through DPPH free-radical scavenging assay [97]. Comparatively high antioxidant activity (88%) was observed for callus tissues grown under yellow light (35–45 μmol/m^2^s), followed by red light-grown callus (80%). When different light/dark regimes were tested, antioxidant activity decreased in the order callus cultures grown under continuous dark > 2 weeks of dark + 2 weeks of light > continuous light [97]. In the dark-grown *A. bracteosa* cell culture, the free-radical scavenging activity correlated with the activity of PAL [94].

### 8.2. Antimicrobial Activity

*A. genevensis* callus extracts exhibited antimicrobial activity against non-pathogenic microorganisms, including *Bacillus subtilis* 205 and 1759, *B. mesentericus*, *Staphylococcus citreus*, *S. aureus* 209, *Escherichia coli* 5009, *E. coli* 205, and *Salmonella typhimurium* 1474 [122]. Later, the authors reported high antimicrobial activity of the aqueous extracts of *A. genevensis* callus of leaf and root origin towards various Gram-positive (*Bacillus subtilis* A1WT; *B. mesentericus* WDCM 1873; *Staphylococcus aureus* WDCM 5233; *S. citreus* WT) and Gram-negative (*Escherichia coli* WKPM M-17; *Salmonella typhimurium* TA 100) microorganisms. The minimal and half-maximal inhibitory concentrations against *E. coli* corresponded to the 70 μg/mL and 140 μg/mL concentration of the extract, respectively [98]. Pathogenic microorganisms, such as *Yersinia pestis*, *Y. enterocolitica*, *Brucella abortus,* and *Bacillus anthracoides,* also showed high sensitivity to water extracts of *A. genevensis* callus cultures [122]. In general, water extracts exhibited higher antimicrobial effects compared to ethanol and methanol extracts.

### 8.3. Biological Activities on the In Vitro and In Vivo Models

Biological activity tests of cell culture extracts or individual compounds in vitro and in vivo are limited to a very few studies.

Teupolioside, a phenylpropanoid glycoside from the cell culture of *A. reptans*, strain IRBN22, was reported to have positive effects on a rat model with induced colitis [146]. Teupolioside treatment reduced diarrhea and body weight loss, and exhibited anti-inflammatory activities. The authors observed amelioration in the disruption of the colonic architecture and a significant reduction in colonic myeloperoxidase activity and malondialdehyde level. They concluded that administration of teupolioside may be beneficial for the treatment of inflammatory intestinal diseases [146].

Verbascoside- and teupolioside-containing extracts from *A. reptans* cell culture accelerated wound healing and demonstrated anti-inflammatory effect in the excision wound model [148]. Furthermore, the extracts were effective inhibitors of chemokine and growth factor expression by cultured human keratinocytes treated with pro-inflammatory cytokines, TNF-alpha, and interferon-gamma [148]. A combination of commercial pollen extract and teupolioside induced a statistically significant improvement of symptoms in patients with Benign prostatic hyperplasia with lower urinary tract symptoms without the development of adverse drug reactions [149].

Aqueous extracts of *A. genevensis* callus cultures were found to be non-toxic on a K-562 suspension cell line of human chronic myeloid leukemia and a human monocytic leukemia cell line 41 [98,122].

### 8.4. Other Uses of Ajuga Cell Cultures

A suspension cell culture of *A. reptans* was suggested as a system for bioconversion of emodin and aloe–emodin—the most common aglycones of 1,8-anthraquinone glycosides, bioactive constituents of *Rhamnus*, *Aloe,* and *Rheum* genera with cathartic and potential anti-cancer activities [102]. The best results with the bioconversion yield near 35–38% for both aglycones were achieved after 48–72 h of contact.

Cell cultures of *A. pyramidalis* Metallica Crispa with varied levels of pigmentation helped in developing analytical methods based on machine vision [150]. Cell lines of the same species and *A. reptans* “Burgundy Glow” were proven to be excellent models for practical laboratory exercises due to their rapid growth and distinct colors. The experiments with differently pigmented cell lines could be used to effectively demonstrate the influence of environment (light wavelength, carbohydrate source) and non-environment (genotype) factors on secondary metabolite accumulation in undifferentiated cell systems [151].

## 9. Comparison of Cell Culture Production Systems with Plants and Differentiated Tissue Cultures

Although major *Ajuga* bioactive compounds can be *de facto* synthesized in undifferentiated cell culture, several authors hypothesized that PEs production in plant tissues may be related to differentiation [21,120]. Furthermore, undifferentiated cell cultures are often morphologically and genetically heterogenous [22]. As a result of dedifferentiation and elimination of the whole plant organization level, the secondary metabolite production may differ between cell cultures, plants, and other cultivation systems, such as somatic embryos and hairy and adventitious root cultures. In *Ajuga*, there are very few studies providing direct comparison of different production systems, including cell cultures, obtained from the same plant source [21,101,104,127]. Therefore, in some cases, the indirect comparisons of cell culture production with plants and organized tissues were compiled using literature data.

### 9.1. Cell Culture Production System Compared to Wild and Greenhouse-Grown Plants

Data presented in Table 2 and Table 3 suggest that callus and suspension cell cultures of *Ajuga* spp. produced a considerably narrower range of secondary metabolites, including PEs, compared to plants (Table 1). The major PEs detected in the cell cultures was 20-hydroxyedysone followed by turkesterone. Iridoids were found in the cell culture of only one out of eight taxa, and the presence of withanolides or neo-clerodane terpenoids was not reported. It is unclear, however, if these data reflect a physiological incapability of the cell cultures to produce a complex mixture of secondary metabolites or the limitations of the analytical methods available to researchers who focused exclusively on PEs. The few examples of direct comparisons between the cell cultures and plants are discussed below.

In *A. reptans,* callus culture produced only four out of seven PEs identified in plants: 20E, polypodine B, 29-norcyasterone, and ajugalactone, while plants also contained 29-norsengosterone, sengosterone, and ajugasterone [101]. The major PEs detected in both plants and the young callus was 20E, and its content in callus cultures increased after 6 years of continuous cultivation and became 4–8-fold higher compared to plants. Cytochrome P450 concentration and ecdysone 20-monooxygenase activity were higher in callus than in roots, stem, and inflorescence of plants, but slightly lower than in plant leaves at vegetative stage [18]. In invertebrates, cytochrome P450-dependent monooxygenases are known to catalyze introduction of hydroxyl groups at position C2 of the core and positions C20, C22, and C25 of the side chain of sterol. Ecdysone 20-monooxygenase catalyzes hydroxylation of ecdysone with the formation of 20E, and analogue of this enzyme has been previously reported in plants and the cell culture [18].

In *A. turkestanica* cell culture, 20E production (up to 0.12%) was higher compared to the leaves and roots of the plants (0.02–0.045%), while the content of turkesterone (0.032–0.036%) was comparable to that in plant roots (0.052%) [105]. By contrast, Cheng et al. [104] found the content of 20E in elicited suspension cell culture (23.6 µg/mg) to be lower than in the shoots of wild plants (37.01 µg/mg). In their work, plants also contained notable quantities of turkesterone (20.41 µg/mg), cyasterone (12.61 µg/mg), and cyasterone 22-acetate (12.91 µg/mg), which were detected in the cell cultures only in trace amounts.

Callus cultures of *A. genevensis* contained 10-methylnonadecane, 1-bromopentadecane and methoxyacetic acid, and 2-tetradecyl ester, while the main components of intact plant extracts were hexacosane, n-hexadecanoic acid, and 2-methoxy-4-vinylphenol [98]. Alanine content in callus tissue of *A. genevensis* (0.217 mg/g) was almost twice as high as its content in plants [122], whereas serine content was similar in plants and callus (near 0.98 mg/g).

The composition of polysaccharides was different between callus and aerial plant parts of *A. turkestanica* [147]. Callus contained a larger portion of water-soluble polysaccharides and a lower quantity of pectin substances compared to plants. Galactose and arabinose predominated in water-soluble polysaccharides of both callus and plant parts. Hemicelluloses of the plant contained high amounts of xylose, while in callus it was only found in trace amounts.

The growth of the pathogenic microorganisms *Yersinia pestis*, *Y. enterocolitica*, *Brucella abortus,* and *Bacillus anthracoides* was inhibited in response to water extracts made of *A. genevensis* callus tissues as well as aerial parts and roots of in vitro-produced mericlones, but the extracts of in vivo plants showed no antimicrobial activity [122].

### 9.2. Cell Culture Production System Compared to In Vitro-Grown Shoots

In vitro-grown shoots were primarily developed as an effective tool for micropropagation of valuable *Ajuga* species [20,56], but they were also reported to contain PEs together with other compounds of pharmacological importance, such as fatty acids, tocopherols, volatile compounds, and carotenoids including lutein [152,153,154,155].

While the cell culture of *A. multiflora* was developed primarily for 20E production [100], in vitro-cultured shoots of this species produced carotenoids lutein, all-E-b carotene, 90-Z-neoxanthin, all-E-violaxanthin, all-E-zeaxanthin, and all-E-b-cryptoxanthin, as well as tocopherols and polyunsaturated FA [152,153]. The amounts of those compounds were higher in the leaves of in vitro plants compared to greenhouse plants [152].

The profiles of volatile compounds differed significantly between cultured cells, in vitro shoots, and leaves of wild plants [30,94,154]. Ali et al. [154] detected 34 volatile compounds in the in vitro-raised shoots of *A. bracteosa* compared to 29 compounds in the cell culture [94]. Both shoots and cell cultures produced substantial amounts of monoterpene hydrocarbons and oxygenated monoterpenes; moreover, sesquiterpenes were also detected in cultured cells [94].

### 9.3. Cell Cultures Production System Compared to Hairy and Adventitious Root Cultures

Similar to the cell culture, hairy and adventitious roots cultured in vitro can serve as excellent models to study mechanisms of PEs biosynthesis [156,157], but they are primarily utilized as biotechnological systems for the production of plant secondary metabolites, including PEs [21,24,158].

Hairy roots of *A. turkestanica* contained 10.5 µg/mg 20E without elicitation, which was slightly higher than the 6.9 µg/mg 20E in untreated cell suspension. The best elicitation treatment for both cell and hairy root culture was 125 µM MeJ, which increased 20E production to a similar level of ca. 23 µg/mg [104]. In the hairy roots of *A. multiflora*, the 20E content (up to 6.4 mg/g) was ten times higher than that measured in the roots of the wild plants (0.6 mg/g) and comparable with 20E content in the cell culture of the same species (up to 6 mg/g) after elicitation with MVA [100]. Hairy roots of *A. bracteosa* elicited with MeJ at optimal concentration (125 µM) produced ca. 6.789 mg/g 20E [26], which was also comparable with the elicited cell cultures of several *Ajuga* species (Table 3).

Hairy roots developed from *A. reptans* were able to produce 0.085–0.15% 20E compared to 0.03% in non-transformed *Ajuga* roots [159], which was slightly lower that in root-originated callus (0.68%) and suspension cell culture (0.43–0.50%) of the same species [101]. Four PEs, namely, 20E, norcyasterone B, cyasterone, and isocyasterone, were identified in different clones of *A. reptans* hairy roots, with 20E content reaching 0.12% [160]. Regenerants developed from these hairy roots retained the capability of 20E production [161]. Hairy roots of *A. reptance* produced 0.21–0.22% 20E when cultured in a turbine-blade fermenter using fed-batch regime [162].

The stability of PEs in hairy roots in the course of continuous cultivation is not clear, although in *A. turkestanica*, some increase in 20E, turkesterone, and cyasterone content was detected at seventh subculture compared to the fourth subculture [104].

Similar to the cell culture of *A. bracteosa*, the adventitious root culture of this species was also capable of producing phenolic and flavonoid compounds and showed high antioxidant capacity [163,164].

Root organogenesis in the callus culture of *A. turkestanica* decreased PEs content [165]. However, shoot morphogenesis on callus cultures and the production of new callus and suspension cell lines from the leaves of the regenerated sprouts allowed the re-establishment of cell lines with enhanced PEs yield [165].

In vitro shoot, cell, and hairy root cultures of *A. turkestanica* produced 20E as a major compound [104]. Among these cultures, only in vitro shoots showed 20E content comparable to wild plants (37.01 µg/mg). Cell suspension accumulated primarily 20E, while hairy roots produced a mixture of 20E, cyasterone, and cyasterone 22-acetate. Turkesterone content decreased in the order wild plants > in vitro shoots > hairy roots > cell culture [104].

In conclusion, isolated root cultures under favorable conditions produce PEs in amounts that are comparable to cell cultures, which implies that PEs accumulation has little dependence on issue differentiation. On the other hand, in several studies, root cultures were capable of producing a mixture of PEs, while 20E was a predominant compound in cell cultures. In both cell and root cultures, MeJ was the most effective as exogenous elicitor.

## 10. Conclusions and Prospects

To date, cell cultures have been developed for eight *Ajuga* taxa, including *A. bracteosa*, *A. chia*, *A. genevensis*, *A. lobata*, *A. multiflora*, *A. pyramidalis*, *A. reptans,* and *A. turkestanica*. Phytoecdysteroids were found in cell cultures of five species: *A. genevensis*, *A. lobata*, *A. multiflora*, *A. reptans,* and *A. turkestanica;* their content varied from trace quantities to amounts comparable or even higher than Pes content in wild or greenhouse plants and hairy root cultures. However, the array of target metabolites detected in the cell cultures was much narrower compared to whole plants. The most abundant PEs in the cell cultures was 20E followed by turkesterone (specifically for *A. turkestanica*) and minor components including polypodine B, 29-norcyasterone, ajugalacton, ajugasterone, and cyasterone 22-acetate. Cell cultures were also capable of producing a large spectrum of other metabolites belonging to different chemical classes: a variety of phenolic and flavonoid compounds, anthocyanins, volatile compounds, phenyletanoid glycosides, a number of iridoids, and some others. Several cell cultures could be used for the production of specific compounds, such as teupolioside, or demonstrated profound antioxidant and antimicrobial activities. Elicitation was proven to be effective for increasing secondary metabolite content, including PEs, in the cell cultures, and MeJ at concentration range of 50–125 µM appeared to be the best option for different cell lines, followed by MVA feeding. Induced mutagenesis was another effective approach to develop a highly productive cell strain. The review of the published experimental studies suggest that *Ajug*a cell cultures possess high potential for the sustainable production of biologically active metabolites of this genus. However, there are several blank spots and challenges that require further research interventions.

First, from 80 species in this genus, only 10% were explored for cell culture development. Hence, it would be of great practical and scientific interest to develop cell cultures from a larger number of *Ajuga* species of high medicinal value and perform their biochemical profiling. This may include the rather comprehensively studied *A. decumbence*, as well as *A. iva*, *A. remota,* and *A. laxmannii,* due to their proven biological activities.

Further, it is worth exploring the potential of the cell cultures for the production of iridoids, neo-clerodane diterpenoids, and withanolides. At the current point, it is unclear whether these compounds had vanished in the cell culture or simply were not detected due to the limitation in analytical methodology. The advanced chemical analysis of the cell culture extracts using modern methods of structural identification will be needed to unleash their full potential for the production of pharmacologically and dietary important substances.

From the practical viewpoint, the bioreactor cultivation of the *Ajuga* cell cultures needs to be developed and optimized for the large-scale production of cell biomass containing the compounds of interest. This may require selection of the highly productive cell strains that are more tolerant of bioreactor conditions, including mechanical damage caused by aeration or mechanical stirring.

Although the developed cell cultures seem to sustain their growth characteristics over the years, the stability of PEs in the course of long-term cultivation may become an issue. Several studies demonstrated that 20E was the only PE remaining in the cell culture after several years of subcultivations, while turkesterone and minor PEs disappeared or reduced to trace amounts. This issue could be potentially resolved by cryopreserving the highly productive young cell lines during the first years of cultivation.

In conclusion, cell cultures of *Ajuga* spp. have great potential for the sustainable production of dry cell biomass, crude extracts, and individual bioactive compounds, which may be used, after proper certification, in pharmacology, cosmetics, or dietary supplement ingredients and reduce the dependence of these industries on wild plant collection.

## Figures and Tables

**Table 1:** Summary of biological activities and chemical composition reported for medicinal *Ajuga* species that were utilized in the cell culture technology *.

Species	Biological Activities Reported **	Phytoecdysteroids	Other Compounds	References
*A. bracteosa*	Antimicrobial (antiviral, antiplasmodial (antimalarial), against hepatitis C, antibacterial), anti-inflammatory (including against arthritis and osteoporosis), cytotoxic, antidiabetic, hepatoprotective, antioxidant, analgesic antidepressant, anticoagulant, anti-cancer, immunoregulatory, insecticidal, cardiotonic, anti-Alzheimer, antihypertensive	20-hydroxyecdysone makisterone A ajugalactone cyasterone 3-epicyasterone 3-*epi*-22-acetylcyasterone	**Withanolides**: bracteosin A and B, ajugin 1, C, D, E, withaferin A **Iridoids**: reptoside, 6-deoxyharpagide **Other sterols**: ß-sitosterol, stigmasterol **Neo-clerodane diterpenoids**: clerodin, 14,15-dihydroclerodin, ivain II, 14,15-dihydroajugapitin, 14,15-dihydro-15-hydroxyajugapitin, ajugarin I, lupulin A, 15-epi-lupulin B, ajubractins A-E, bracteonin-A, 3-*epi*-caryoptin, 3-*epi*-14,15-dihydrocaryoptin, 15-hydroxyajubractin C, 14-hydro-15-hydroxyajugachin A **Other compounds**: pyrocatechol, resorcinol, catechin, gallic acid, chlorogenic acid, caffeic acid, syringic acid, p-coumaric acid, ferulic acid, vanillic acid, coumarin, sinapinic acid, trans-cinnamic acid, rutin, quercetin, kaempferol, 3,4′-dihydroxy-3,6,7-trimethoxyflavone, 7-hydroxy-3,6,3′,4′-tetramethoxyflavone, ajuganane, bis(2S-methylheptyl) phthalate, heptacos-3-en-25-one, bractic acid, bractin A and B Essential oils	[2,4,6,26,28,29,30,31,32,33,34,35,36,37,38,39,40,41,42]
*A. genevensis* (including synonym *A. pyramidalis*)	Antioxidant, antimicrobial (including antifungal), anti-inflammatory, antiproliferative, antitumor	No information	**Neo-clerodane diterpenoids:** ajugavensins A–C, ajugapyrin A (in *A. pyramidalis*) **Iridoids**: harpagide, aucubin, catalpol, harpagoside, 8-O-acetyl-harpagide **Other compounds:** coumaroyl glucoside and its isomer, caffeic acid, p-coumaric acid, ferulic acid, rosmarinic acid, oleanolic acid, maslinic acid, hyperoside, quercitrin, quercetin glucuronide, apigenin, apigenin-C-hexoside-C-pentoside, forsythoside A, luteolin and its derivatives, campesterol	[43,44,45,46,47,48,49]
*A. chamaepitys*	Anti-inflammatory, antioxidant, antimicrobial, antitumor, anti-arthritic, antimalarial, wound-healing, activity against ulcerative colitis	20-hydroxyecdysone makisterone A cyasterone	**Iridoids:** 8-O-acetylharpagide, harpagide, aucubin, catalpol, harpagoside, ajugoside, reptoside, 5-O-β- D -glucopyranosyl-harpagide, 5-O-β-D-glucopyranosyl-8-O-acetylharpagide, asperulosidic acid, deacetylasperulosidic acid **Other compounds:** ferulic acid, isoquercitrin, rutin, quercitrin, luteolin, 53 volatile compounds (major–β-pinene, ethyl linoleate, germacrene D, kaurene, (E)-phytol, γ-terpinene, limonene), acteoside, chrysoeriol 7-O-glucopyranoside, pigenin 7-O-rhamnopyranoside	[43,50,51,52,53,54,55]
*A. multiflora*	Muscle-protective, cytotoxic against murine leukemia tumor, antibacterial, pesticidal	20-hydroxyecdysone 29-hydroxyprecyasterone cyasterone makisterone A	**Iridoids:** 8-O-acetylharpagide, harpagide **Other compounds:** apigenin, apigenin 7-glucuronide, bis(2-ethylhexyl) phthalate, carotenoids (including E-lutein, all-E-β-carotene, 9′-Z-neoxanthin, all-E-violaxanthin, all-E-zeaxanthin, all-E-β-cryptoxanthin), fatty acids (including linoleic, linolenic, and palmitic)	[2,56,57,58,59,60]
*A. reptan* *s*	Antioxidant antimicrobial (antibacterial, antifungal), anti-inflammatory, androgenic, neuroprotective, antipredatory	20-hydroxyecdysone ajugalactone ajugasterone A, B cyasterone 29-norcyasterone 29-norsengosterone 2-acetyl-29-norcyasterone 3-acetyl-29-norcyasterone sengosterone 24,25-dehydroprecyasterone 20-hydroxyecdysone 22-acetate 20-hydroxyecdysone 25-acetate (viticosterone E) breviflorasterone reptanslactone A and B sendreisterone polypodine B reptansterone viticosterone E 28-*epi*-sengosterone 5,29-dihydroxycapitasterone, 2-dehydroajugalactone, 3-dehydroajugalactone	**Iridoids:** reposide, ajugoside, ajugol, 8-O-acetylharpagide, harpagide, ajureptoside, aucubin, catalpol, harpagoside **Sterols:** clerosterol, 22,23-didehydroclerosterol, campesterol, ß-Sitosterol **Neo-clerodane diterpenoids**: ajugareptansin, ajugareptansone A, ajugareptansone B, ajugachin A, ajugavensin A, ajugorientin (3β-hydroxyajugavensin B), 14,15-dehydroajugareptansin, 3α-hydroxyajugamarin F4, areptin A, areptin B, ajugatansin A1, ajugatansin B1, ajugatansin D1, ajugareptone **Other compounds:** chlorogenic acid, caffeic acid, luteolin, luteolin-7-O-glucoside, apigenin, p-Coumaric acid, ferulic acid, leucoseptoside A, verbascoside, cistanoside A, forsythoside A, echinocoside, caffeoyl glucose, isoquercitrin, rutin, quercitrin	[2,7,8,38,44,61,62,63]
*A. turkestanica*	Antiproliferative, antimicrobial (antibacterial), antioxidant, hypoglycemic, hypolipidemic, anabolic, hepatoprotective, increase in protein synthesis in skeletal muscle and in liver, increase signaling in aged skeletal muscles, muscle strength improvement, stimulation of aquaporins–human skin hydration, erythropoesis-stimulating	20-hydroxyecdysone cyasterone 11-hydroxy-cyasterone 11-hydroxy-sidisterone 11-hydroxy-Δ24-capitasterone ajugalactone 22-acetylcyasterone turkesterone ajugasterone B atrotosterone C abutasterone ajugasterone C 25-hydroxy-atrotosterone A 25-hydroxy-dacryhainansterone turkesterone 22-acetate 22-oxo-turkesterone turkesterone 22-acetonide	**Neo-clerodane diterpenes:** ajugapitin (clerodendrin D), chamaepitin, ajugachin B, lupulin A, 14,15-dihydroajugachin B, 14-hydro-15-methoxyajugachin B **Iridoids:** harpagide, 8-O-acetylharpagide	[2,7,17,58,64,65,66,67,68]

* Information is presented selectively for those species in which development of the cell cultures was reported: *A. bracteosa, A. chia* (synonym of *A. chamaepitys*), *A. genevensis, A. lobata, A. multiflora, A. pyramidalis* (synonym of *A. genevensis*), *A. reptans.* ** According to in vitro and in vivo studies.

**Table 2:** Examples of nutrient media and explant sources used for cell culture induction (I) and maintenance (M) in various *Ajuga* species.

Species	Explant Source	Culture Type	Medium Composition	Reference
*A. bracteosa*	Leaves Petioles Internodes	Callus Callus Callus	**I**: MS + 5.0 mg/L BA **I**: MS + 2.0 mg/L BA + 3.0 mg/L IAA **I**: MS + 2.0 mg/L BA + 5.0 mg/L NAA	[96]
*A. bracteosa*	Leaves of in vitro plantlets	Callus	**I**: MS + 2.0 mg/L BA + 1.0 mg/L 2,4-D	[95]
*A. bracteosa*	Sterile hypocotyls	Callus, suspension	**I, M**: MS + 1.0 mg/L BA	[94]
*A. bracteosa*	Sterile hypocotyls	Callus	**I**: MS + 1.0 mg/L TDZ + 0.5 mg/L NAA	[97]
*A. genevensis*	Leaves and roots	Callus	**M**: MS + 2.0 mg/L glycine + 2.0 mg/L IAA + 0.2 mg/L kinetin	[98]
*A. lobata*	Roots	Callus, suspension	**I**: MS + 1 mg/L BA + 0.4 mg/L 2,4-D **M**: MS + 0.4 mg/L 2,4-D for callus and 0.4 mg/L 2, 4-D + 0.5 mg/L BA for suspension	[99]
*A. multiflora*	Leaves	Callus, suspension	**I**: MS + 0.2 mg/L BA + 0.2 mg/L kinetin + 0.4 mg/L 2,4-D) **M**: MS + 0.4 mg/L 2,4-D for callus and 0.6 mg/L 2,4-D for cell suspension	[100]
*A. pyramidalis*	In vitro developed leaves	Callus	WPM + 100 μM Fe as FeNa_2_EDTA + rose vitamins + 0.1 g/L myoinositol, + 0.15 g/L PVP + 0.05 g/L L-ascorbic acid + 2.26 μM 2,4-D + 3.49 μM kinetin	[93]
*A. reptans*	Roots	Callus, suspension	**M** (callus): MS + Staba vitamins + 100 mg/L inositol. **M** (suspension): MS + Staba vitamins + 100 mg/L mesoinositol + 1 mg/L NAA + 2 g/L PVP	[101]
*A. reptans*	Not specified	Callus, suspension	**M**: MS + Staba vitamins + 1 mg/L 2,4-D + 0.2 mg/L BA + 100 mg/L mesoinositol + 1 mg/L NAA + 2 g/L PVP.	[18]
*A. reptans*	Seeds	Callus, suspension	B5 (Gamborg) + 1 mg/L NAA + 1 mg/L kinetin + 0.2 mg/L 2,4-D	[102]
*A. turkestanica*	Leaves	Callus	**M**: MS + 100 mg/L meso-inositol + 0.4 mg/L thiamine HCl	[103]
*A. turkestanica*	Leave of in vitro plants	Callus, suspension	**I, M**: B5 (Gamborg) + 2.31 M 2,4-D	[104]
*A. turkestanica*	Ovary tissues	Callus, suspension	**I, M** (callus): MS + 1 mg/L NAA + 0.002 mg/L TDZ **I, M** (suspension): 1/2MS + 1 mg/L NAA + 0.0002 mg/L TDZ	[105]

MS—Murashige and Skoog medium [106], B5—Gamborg medium [107], WPM—woody plant medium [108]. Staba vitamins: 0.5 mg/L folic acid, 0.5 mg/L riboflavin, 1 mg/L biotin, 1 mg/L calcium pantothenate, and 0.0015 mg/L cobalamin [109]; rose vitamins: 0.5 mg/L thiamine-HCL, 13.9 mg/L ferrous sulfate heptahydrate, 18.6 mg/L pyridoxine-HCL, 0.5 mg/L nicotinic acid, 0.5 mg/L glycine [110]. NAA—α-Naphthalene acetic acid, BA—6-benzylaminopurine, 2,4-D—2,4-dichlorophenoxyacetic acid, IAA—indole-3-acetic acid, TDZ—thidiazuron, PVP—polyvinylpyrrolidone.

**Table 3:** Biosynthesis of phydoecdysteroids in cell cultures of *Ajuga* spp. and elicitation conditions for maximum PEs yield.

Species	Culture Type	Extract Analyzed	Compounds Identified	Content and Optimum Elicitation Treatment	Reference
*A. genevensis*	Leaf-originated callus	Water, methanol, or ethanol extracts	Non-identified ecdysteroids	-	[122]
*A. lobata*	Cell suspension	Not available	20-Hydroxyecdysone	Elicitation: 0.1 mg/L ABA *	[123]
*A. lobata*	Root-originated cell suspension	Methanol extracts	20-Hydroxyecdysone	Up to 12.75 mg/gDW Elicitation: 10 mg/L MVA (day 1) + 50 μL/L α-Pinene (day 1) + 80 mmol/L SNP (day 7)	[99]
*A. lobata*	Cell suspension	Not available	20-Hydroxyecdysone	Up to 3.53 mg/gDW Elicitation: 100 μmol/L MeJ	[124]
*A. lobata*	Cell suspension	Methanol extracts	20-Hydroxyecdysone	Up to 7 mg/gDW Elicitation: 0.15 mg/L ABA	[125]
*A. multiflora*	Leaf-originated cell suspension	Methanol extracts	20-Hydroxyecdysone	Up to 6 mg/gDW Elicitation: 10 mg/L MVA	[100]
*A. reptans*	Callus	Methanol extracts	20-Hydroxyecdysone	0.121%	[18]
			Polypodine B	0.001%	
			29-Norcyasterone	0.002%	
			29-Norsengosterone	0.001%	
*A. reptans*	Root-originated callus and cell suspension	Ethanol extracts	20-Hydroxyecdysone	0.68% in callus, 0.43–0.50% in suspension	[101]
*A. reptans*	Callus	Methanol extracts	20-Hydroxyecdysone, Polypodine B, 29-Norcyasterone, Ajugalactone, Ajugasterone	Elicitation: 2.5 mM MnSO_4_ *	[121]
*A. turkestanica*	Leaf-originated callus and cell suspension	Methanol followed by petroleum ether and ethyl acetate extraction	20-Hydroxyedysone,	23.6 µg/mg extract (20E) Elicitation: 125 μM MeJ	[104]
			Turkesterone Cyasterone Cyasterone 22-acetate	Trace amounts Trace amounts Trace amounts	
*A. turkestanica*	Ovary-originated callus	Methanol extracts	20-hydroxyedysone	0.1–0.12%	[105]
			Turkesterone	0.032–0.036%	
*A. turkestanica*	Not specified	Methanol followed by butanol extracts	20-Hydroxyedysone	0.029%	[113]
*A. turkestanica*	Ovary-originated callus	Methanol extract	20-Hydroxyedysone	0.035%	[126]
*A. turkestanica*	Mutant callus (N-NMU treated)	Methanol extract	20-Hydroxyedysone Turkesterone	0.2% Not given	[119]
*A. turkestanica*	Leaf-originated callus and cell suspension	Water-ethanol extracts	20-Hydroxyecdysone	Up to 2.5 mg/gDW	[120]
			Turkesterone	0.04–0.05 mg/gDW	

MeJ—methyl jasmonate, ABA—abscisic acid, MVA—mevalonic acid, 20E—20-hydroxyedysone. * Content not given or information is not available.

**Table 4:** Compounds of different chemical classes found in *Ajuga spp.* cell cultures.

Species	Culture Type	Extract Analyzed	Compounds Identified	Content and Optimum Elicitation Treatment	Reference
*A. bracteosa*	Hypocotyle-originated callus and cell suspension	Acetone-water extracts for total phenolics and flavonoids, hydro-distillation through a Clevenger-type apparatus for volatiles	Total phenolics, total flavonoids 29 volatile compounds including: β-Pinene	7.0 mg GAE/g DW 3.8 mg QE/g DW Elicitation: 0.5 mg/L MeJ 2.1–9.5%	[94]
			β-Ocimene	1.4–8.3%	
			1-Terpinene-4-ol	5.8–9.6%	
			Caryophyllene	1.3–6.2%	
			β-Farnesene	0.82–7.8%	
			Myrtenal	2.2–8.4%	
			Citronellyl acetate	2.1–7.3%	
			Caryophyllene oxide	1.5–5.5%	
			β-Elemene	2.2–8.8%	
*A. chia* (accepted name *Ajuga chamaepitys* subsp. *chia*)	Leaf-originated callus	Hexane extracts	Neutral lipids, palmitic, stearic, oleic, linolenic, linoleic, arachic fatty acids	0.89–1.48%	[145]
*A. genevensis*	Leaf-originated callus	Water, methanol, or ethanol extracts	Alanine Serine	0.217 mg/gDW 0.98 mg/gDW	[122]
*A. genevensis*	Leaf- and root-originated callus	Water, ethanol, or methanol extracts	10-Methylnonadecane	57.0%	[98]
			Methoxyacetic acid 2-tetradecyl ester	17.75%	
			1-Bromopentadecane	14.55%	
*A. genevensis*	Leaf- and root-originated callus	Hexane extracts	Neutral lipids, palmitic, stearic, oleic, linolenic, linoleic, arachic fatty acids	0.65–0.96%	[145]
*A. pyramidalis*	Leaf-originated callus and cell suspension	Methanol-HCl extracts	Ferulic acid	138 mg/100 gFW	[93,137]
			Anthocyanins	up to 17–42 mg/100 gFW	
*A. reptans*	Leaf-originated cell suspension	Ethanol-water extract	Teupolioside	4 g/L	[146]
*A. reptans*	Flower-originated callus and cell suspensions	Methanol-acetic acid-water extracts	Cyanidin- and delphinidin-based anthocyanins	1–2.5% DW	[111,112,139,140,142]
*A. turkestanica*	Leaf-originated callus	Methanol extracts	Leonoside A, lavandulifolioside	Not given	[103]
*A. turkestanica*	Ovary-originated callus	Water, oxalate buffer, alkali, precipitation with alcohol	Polysaccharides: WSPS PS HMC A and B	7.2% 7.0% 13.1%	[147]
*A. turkestanica*	Mutant callus (N-NMU treated)	Methanol extract	Harpagide, 8-O-Ac-harpagide	Not given	[119]
*A. turkestanica*	Ovary-originated callus	Ethanol, water, oxalate extracts	Polysaccharides: SWPS PS	Up to 13.7% Up to 5.7%	[126]

GAE—gallic acid equivalent, QE—quercetin equivalent, MeJ—methyl jasmonate, WSPS—water-soluble polysaccharides, PS—pectin substances, HMC A and B—hemicellulose A and B, FW—fresh weight, DW—dry weight.

## Data Availability

All data used in this study were gathered from open literature sources or scientific journals available under institutional subscription.
